# Strategies to prevent HIV transmission among heterosexual African-American women

**DOI:** 10.1186/1475-9276-4-4

**Published:** 2005-03-17

**Authors:** E James Essien, Angela F Meshack, Ronald J Peters, GO Ogungbade, Nora I Osemene

**Affiliations:** 1 The HIV Prevention Research Group. College of Pharmacy, University of Houston, 1441 Moursund Street, Houston, Texas 77030. USA; 2 Center for AIDS Research, Baylor College of Medicine, Houston, USA; 3 WHO Center for Health Promotion and Prevention Research, University of Texas School of Public Health, Houston, Texas 77030, USA; 4 College of Pharmacy. Texas Southern University. Houston, Texas 77004., USA

**Keywords:** African Americans, women, HIV, AIDS, Risk behaviors, Intervention.

## Abstract

**Background:**

African-American women are disproportionately affected by HIV, accounting for 60% of all cases among women in the United States. Although their race is not a precursor for HIV, the socioeconomic and cultural disparities associated with being African American may increase their risk of infection. Prior research has shown that interventions designed to reduce HIV infection among African-American women must address the life demands and social problems they encounter. The present study used a qualitative exploratory design to elicit information about strategies to prevent HIV transmission among young, low-income African-American women.

**Methods:**

Twenty five low income African American women, ages 18–29, participated in five focus groups of five women each conducted at a housing project in Houston, Texas, a large demographically diverse metropolitan area that is regarded as one of the HIV/AIDS epicenters in the United States. Each group was audiotaped, transcribed, and analyzed using theme and domain analysis.

**Results:**

The participants revealed that they had most frequently placed themselves at risk for HIV infection through drugs and drinking and they also reported drug and alcohol use as important barriers to practicing safer sex. The women also reported that the need for money and having sex for money to buy food or drugs had placed them at risk for HIV transmission. About one-third of the participants stated that a barrier to their practicing safe sex was their belief that there was no risk based on their being in a monogamous relationship and feeling no need to use protection, but later learning that their mate was unfaithful. Other reasons given were lack of concern, being unprepared, partner's refusal to use a condom, and lack of money to buy condoms. Finally, the women stated that they were motivated to practice safe sex because of fear of contracting sexually transmitted diseases and HIV, desire not to become pregnant, and personal experience with someone who had contracted HIV.

**Conclusion:**

This study offers a foundation for further research that may be used to create culturally relevant HIV prevention programs for African-American women.

## Background

Despite the impressive strides that have been made by behavioral scientists in developing culturally sensitive HIV intervention programs for minority populations in the United States, HIV infection continues to be a major public health problem and is increasingly affecting minority populations, persons infected through heterosexual contact, the poor and women [1]. African-American women are one group disproportionately affected by HIV [1,2]. Although they constitute only 13% of the female population, they account for 68% of all HIV cases among women in the United States [3-5] and AIDS is the leading cause of death among African-American women, aged 25 to 44 years [4,6]. The rate of HIV infection among African-American women is estimated to be four times higher than the rate for Latinas and more than nineteen times higher than the rates for Anglo women [7]. The disproportionate rate is further amplified among African-American women who use drugs [2].

The main exposure categories for all women in the United States is heterosexual contact accounting for approximately 40% of all new AIDS cases among this group [2,5]. This is followed by injection drug use that constitutes 25% of all new cases of AIDS among women. Among African-American women, 42% of AIDS cases are attributed to personal injection drug use while 38% are attributable to heterosexual transmission [8]. These behaviors often occur within the same context. From 1995 to 1999, mortality from AIDS has decreased more among men than women and among Whites and people of higher income status. However, the percentage decrease during the same time period was lowest among African-American women and women from the south^1^, suggesting a need for an effective HIV prevention program for these women.

Aside from heterosexual contact and injection drug use, depression, physical and sexual abuse, and lack of condom negotiation skills are some of the psychosocial determinants of HIV risk behaviors among women [5]. Drug use, violence and depression have been described as a tripartite of risk factors that appear to have a profound influence on HIV risk and HIV infection among African-American women [8]. For example, crack cocaine use has had a devastating effect on the African-American community and appears to increase the likelihood of riskier sexual behavior as the amount of crack use increases [9]. In addition, conditions of poverty and homelessness are closely related to trading sex for drugs, a condition that affects many crack cocaine users and one that increases HIV risk [10].

While African-American women may not be placed at risk of HIV infection because of their race and ethnicity, St. Lawrence, Eldridge, Reitman, Little, Shelby, and Brasfield [11] note that race and ethnicity may be a reflection of the socioeconomic and cultural disparities that are associated with HIV transmission. According to Sanders-Philips [12], an understanding of the socioeconomic dynamics of HIV transmission among African-American women and incorporating this information into HIV prevention programs could significantly enhance HIV prevention efforts for these women.

An array of socioeconomic and cultural factors exacerbate high-risk behaviors that place African-American women at risk for HIV infection [12,13], the most notable being the role of poverty. Although poverty in itself is not a precursor for HIV infection, several studies have established a direct link between low socioeconomic status and AIDS incidence [14-17]. For many African-American women, changing HIV-risk-related behavior is difficult because, daily, they deal with problems of poverty by engaging in sex for drug exchanges, prostitution, violence, and powerlessness in negotiating safer sex practices in relationships with African-American men [12,13]. It has been suggested that HIV risk-reduction programs for African-American women must address the life demands and social problems that these women face including poverty, alcohol and drug use, and other cultural and contextual issues that influence the role of women in safer sex decision making in the African-American community [11,18]. Research evidence has shown that for interventions to be effective among ethnic minority populations, they must be presented in a socio-cultural context as well as have gender specificity and a sound theoretical framework [19-21]. To date, only a limited number of studies have explored the impact of these variables on the decision to practice safe sex among African-American women, and the role that they play in developing culturally relevant HIV prevention interventions.

Individual and small group behavior change techniques have long been used in HIV prevention interventions for women and have resulted in increased condom use by inner-city women in primary health care settings, mental health clinics, and among women living in economically disadvantaged neighborhoods [4]. Community-level interventions have been used less frequently, yet they are needed to disseminate health promotion messages that influence individual behavior change and strengthen social norms to support and reinforce such change. Lauby and colleagues [4] reported that a two-year, large-scale community-based intervention significantly impacted partner communication about condom use and attempts to get a main partner to use condoms. Their research highlights the effectiveness of reaching large numbers of women and changing their condom-use behaviors regarding communication with a main sex partner. Safer sex requires both partners' consent and often the male in risky sexual situations is resistant [2]. Theall, et al [5], examined the factors associated with HIV seropositivity among African-American women, aged 18 to 59 years, who were active crack cocaine users and/or injection drug users and found that an inability to say no to male sex partners was the strongest predictor of positive serostatus and as a result, skills building for negotiating and communicating safer sex practices is needed in prevention programs.

Several HIV interventions have been developed to promote condom use and enhance sexual communication skills among African American women. The most promising interventions have been programs that are based on social cognitive principles. However, Kalichman et al [21] notes that HIV prevention programs that are based on social cognitive principles and proven to be effective in the scientific literature have not been widely utilized in community settings because of their dependence on expert interventionists for implementation in face-to-face formats, making them difficult to transfer to community-based organizations. In contrast, social cognitive theory principles applied to HIV prevention can be delivered effectively by videotapes and community-based organization personnel with minimal training in skills building techniques. The rationale for using videotapes as part of an HIV intervention delivery system is provided by the emerging literature which demonstrates the feasibility of this medium in changing high risk sexual behaviors [21,22].

Using constructs from social cognitive theory, the health belief model and theory of reasoned action, Roye & Hudson [22] conducted a study to assess the impact of a culturally appropriate videotape-based intervention on condom use among urban adolescent women who use contraception. The study showed that the videotape based intervention promoted favorable changes in sexual behaviors. Similarly, Kalichman et al [21] tested the efficacy of a culturally sensitive HIV prevention intervention for African-American women by randomly assigning African-American women to three intervention conditions: a single session public health service videotape intervention that provided HIV information delivered by two white women; a second videotape intervention that provided the same information but delivered by an African-American woman; and a third intervention module that was similar to the second but with the addition of culturally relevant materials. The women who received the intervention that used culturally relevant materials reported in follow-up assessments an increase in antibody testing and requests for condoms. Taken together, these studies demonstrate that culturally sensitive videotape-based HIV prevention interventions may be effective in changing high risk sexual behaviors.

The research presented here results from qualitative studies conducted among African-American women in Houston, Texas, to elicit information that could be used to develop an HIV prevention intervention for similar populations. The research had two overarching purposes: to examine the sociocultural contexts of sexual risk taking among African-American women and to determine how a videotape-based HIV prevention intervention could be tailored so that it is effective in preventing HIV transmission among African-American women.

## Methods

### Design

This study utilized a qualitative exploratory design to elicit information about strategies for preventing HIV transmission among African-American women. Twenty five low income African-American women, aged 18–29, participated in five focus groups of five women each conducted at a housing project in Houston, Texas. Houston, Texas, a large demographically diverse metropolitan area, was selected as the study site because of its distinction as a leading HIV/AIDS epicenter in the United States [6]. The housing complex was targeted for convenience sampling and because it is located within the same predominantly low-income African-American community as the research institution. Approval for the study was obtained from the relevant university Committee for the Protection of Human Subjects.

### Procedure

Focus group participants were recruited by displaying posters and flyers at strategic locations in the housing complex identified by the project manager. The flyer listed the study inclusion criteria: African-American heterosexual female, aged 18 to 29, self-reported unprotected vaginal intercourse with two or more partners or injection drug using partner in the last six months, or having been diagnosed or treated for a sexually transmitted disease in the past year. The flyer listed a university telephone number that prospective participants could call to obtain additional information about the study and/or to schedule their participation in a group. A trained research assistant confirmed the caller's eligibility. The study participants were recruited using a convenience sampling approach and study participants encouraged their friends to enroll in the study. Of the 89 prospective participants who contacted the university, 42 agreed to participate. Of that number, seventeen individuals did not appear on the scheduled day and thus 25 individuals formed the study sample. The groups were conducted at the housing project's clubhouse by a facilitator, slightly older than the target group, trained in focus group methodology and having approximately seven years' experience conducting groups with low-income African-American women. The facilitator was assisted by a trained research assistant who took notes to ensure that pertinent information not captured by the audiotape was obtained and to record major points and items discussed.

Participants were told that their involvement would help researchers and clinicians to learn about what they feel is important and better ways of helping the African-American community to combat HIV and AIDS. They were informed that their involvement was voluntary, that they could refuse to answer any question, and that they could cease participation at any time without penalty. Agreement was also obtained to audiotape the session. Prior to the start of each group, active written informed consent was obtained from each participant. The facilitator discussed with participants the issue of confidentiality of the information discussed at the meeting. Because some of the women were familiar with one another as a result of residency within the same housing complex, the facilitator ensured that all women were aware that what was discussed during the session should not leave the session at its conclusion. They were advised that they would receive a $25 mall gift certificate and condoms as incentives for their participation. Respondents were also advised that the tapes, which were anonymous, would be destroyed following transcription and checking. All questions as well as the informed consent were provided in English.

### Data Collection

Semi-structured and open-ended questions were used to elicit information from the participants based on our research interest in determining perceptions of AIDS as a threat to African-American communities, barriers and facilitators to safer sex practices, characteristics of past situations which have placed persons at risk for HIV infection, the role of alcohol and drugs in creating high-risk sexual situations, suggestions on ways to enhance the saliency of AIDS in the African-American community and on how HIV intervention videotapes could be produced to achieve maximum levels of interest [See Table 1].

**Table 1:** Focus group guide questions

1. Do you perceive AIDS as a threat to the African American community and why?
2. What are the perceived roles of women in heterosexual relationships in the African American community?
3. What are the expectations for personal and sexual responsibilities for contraception and sexually transmitted diseases prevention among African American women?
4. What situations have placed you at risk for HIV infection in the past?
5 How have alcohol and drug use placed you at risk of HIV infection?
6. What are the things that motivate you to practice safe sex?
7. What are the things or barriers that prevent you from practicing safe sex
8. Why do you think that AIDS is spreading so rapidly in the African American community?
9. What information do you think we need to include in a videotape developed to train African Americans about HIV prevention that will encourage them to watch the videotapes?
10. Do you have any other suggestions on how AIDS can be prevented in the African American community?
11. What can we do to get people to sign up for focus groups such as this one and also get them to participate in HIV/AIDS training programs?
12. What can we do to make these training programs most useful to you?

Focus group questions were generated in three stages. The first involved conducting interviews with six key informants [four public health researchers, one pharmacist, and one nurse experienced in HIV/AIDS prevention among African Americans] to generate working hypotheses on facilitators and barriers to HIV prevention and program messages and methods. The hypotheses formulated were then tested in interviews with women similar to members of the target population to reshape the hypotheses and continued until no new information emerged. The resulting hypotheses were used to generate questions that were then field tested with members of the target population. At the end of the first group, additional changes were made as needed to the way in which questions were asked not to elicit different information, but rather to add clarity for the participants.

The resulting focus group interviews from which this research is reported were conducted using a guide consisting of a written list of questions and probes. McCracken [23] has highlighted the advantages of utilizing such a guide including to ensure that all areas of interest are covered and to focus the researcher's attention on listening to the informants, thereby enabling a better understanding of their lines of thought and possibly, unanticipated explanations of the concepts. The duration of each group was about two hours. The focus groups were transcribed at the completion of all groups. Because the questions were carefully crafted and the purpose of the groups was to promote self-disclosure and to generate ideas and perceptions about HIV/AIDS in the African-American community, any idea that emerged was considered valid and not subject to verification by the research team.

### Data analysis

Data analysis was performed according to the standard grounded theory approach of Glaser and Strauss [24]. The relatively unclear understanding of the sociocultural contexts of HIV sexual risk taking among African-American women made a qualitative analysis particularly useful. Codes and categories were developed by doing a line-by-line analysis of the participants' transcripts [25] and identifying the emerging themes. The thematic concepts representing ideas expressed by a majority of the members of three or more focus groups were characterized as a domain and are reported below.

## Results

### Study participants

The study sample consisted of 25 women, ages 18 to 29. The mean age was 24 years. Among the 25 women in the sample, two had completed some post-secondary education and the remainder had completed eighth or ninth grade. Twenty of the women were unemployed and most of these women identified themselves as homemakers. Of the remaining five, the most frequently cited source of employment [n = 4] was the medical field [nursing or medical assistant]. Two identified themselves as HIV positive. None of the respondents had health insurance. The names used for the quotes given in this report are pseudonyms.

### Risk situations for HIV transmission

The situational determinants of HIV risk taking and their impact on HIV/AIDS prevention behaviors and education programs were examined. The rate of HIV infection and AIDS is higher among African-American women in Houston and nationally [6] and African-American women are among the poorest of racial/ethnic minorities [26]. Although poverty itself is not a precursor for HIV infection, it does lead to several psychosocial factors that may place African-American women at higher risk for infection. The present population revealed that they had most frequently placed themselves at risk for HIV infection through drugs and drinking and they also reported drug and alcohol use as important barriers to practicing safer sex. Although these behaviors may not be directly linked to poverty, it is reported that people who are oppressed will often turn to substances as a way of coping with daily life [27]. The women also reported that the need for money and having sex for money to buy food or drugs had placed them at risk for HIV transmission.

Comprehension of the situations explicated by low-income African-American women that place them at risk for HIV is of critical importance when developing programs that address HIV risk reduction. Most women had placed themselves at risk of transmission through drug use or needle sharing and having unprotected sex. The sexual activity took place in some instances in exchange for drugs or money and to purchase basic necessities such as food for their children. Other women stated that they had been placed at risk of HIV transmission because they believed they were in monogamous relationships but later learned their partners had been unfaithful.

Edith, a 27 year old volunteer, described the situations that have placed her at risk for HIV in the past year:

When you are on drugs and then you are drinking, it impairs your senses and you don't use common sense or knowledge of what you are doing. You just get caught up in the moment.

Mary, a 26 year old homemaker explains:

Having sex period is a risk. When you can't feed your kids and you need money. When you go sleep around and have sex and they're not using condoms. Cause some of them say they don't use them and some of them say they don't want to use them. They have all kinds of excuses not to use them.

Celia, a 25 year old medical worker, described how she was placed at risk of HIV infection:

I married a man, not really knowing him, and he was sleeping with a lot of women, and sleeping with me unprotected. Yeah, right after we got married, he told me he wasn't going to cheat on me no more and when I found out some of the women he was cheating on me with, I knew that they always stay at the doctor cause something always be wrong with them. I was pregnant and hoping that nothing was wrong with me.

Related to risk for HIV, the participants were asked to discuss why HIV is so prevalent within their [the African-American] community. Unprotected sex, lack of awareness, lack of medical access, and early sexual initiation were most frequently mentioned. When asked why HIV is spreading so rapidly in the African-American community, unprotected sex was stated most often. Lack of knowledge or awareness about prevention was also frequently stated. However, drug use was not highly ranked and was infrequently stated compared to women's beliefs that lack of access to medical care and non-priority of health as well as early sexual initiation and feelings of invincibility among young people were more significant contributors to HIV's rapid spread among African Americans.

Betty, a 25 year old homemaker said:

I think it's spreading around so fast because people that have AIDS just constantly have sex and they pass it on and pass it on and pass it on.

Celia expressed her agreement and added:

Everybody's sleeping with everybody. The women are sleeping with women; the men are sleeping with men and then they sleeping with each other. Ménage á trios or threesomes, foursomes, they popping Ecstasy pills, just partying hard, they smoking marijuana, they speed balling, they taking downers, handlebars. Uh, I don't know, just really not caring and so they're not even going to the doctor to see if anything is wrong with them. They're just continually sleeping and not stopping to take care of their own bodies, to make sure that they are okay.

Kelli, a 24 year old operator stated:

Well, first of all because we're uneducated. And we're just unconcerned. Like they say, you have to do what you have to do to get what you need, and if it's one of them five minute things, they ain't thinking about no condom.

Kim, a 24 year old cashier said:

I don't know – people feel like they can't get it. Maybe they feel like it just can't get to them. They feel like it can get to other people, but it can't get to them.

Although the women related their individual risk for HIV infection to drugs and alcohol, they did not associate drug and alcohol use with the rapid spread of HIV within the overall African-American community.

### Barriers to safe sex practices

When asked to name situations that have prevented them from protecting themselves against HIV infection, the reasons given were much the same as when they were asked about situations that have placed them at risk for HIV infection. About one-third of the women named drug and alcohol use as responsible for them not taking needed precautions. Surprisingly, about one-third of women stated that their barrier was their belief that there was no risk based on their being in a monogamous relationship and feeling no need to use protection, but later learning that their mate was unfaithful. Other reasons given were lack of concern, being unprepared, partner's refusal to use a condom, and lack of money to buy condoms.

Chaka discussed how drug and alcohol use were barriers to safe sex practices for her:

Working in the club, you drink and if you intensify that with drugs, you don't' know who you're going home with. You don't know who been to your house and what they have done to you on account of you being high, on account of you being drunk or you done overindulged in either or and it's just not a good feeling.

Charlie stated that she was placed at risk of contracting HIV by her spouse:

I married a man not really knowing him and he was sleeping with a lot of women and then sleeping with me unprotected.

Vicki, an 18 year old high school student said:

Like I said, not having the money to go and buy the protection. That's going to prevent you from preventing it.

In this culture, particularly among poorer uneducated women, men may play a more domineering role over women [28]. There is also a misconception among African-American men and some women that condom use reduces the sensation produced during sexual intercourse [29]. Some of the women in this group reported that their partners had refused to use a condom.

### Facilitators to safe sex practices

The women were motivated to practice safe sex because of fear of contracting sexually transmitted diseases and HIV, desire not to become pregnant, and personal experience with someone who had contracted HIV. Over one-third of women acknowledged that fear of contracting sexually transmitted infections including hepatitis and HIV motivated them to practice safe sex. A participant stated:

Every disease that's in the book that's not curable is enough to scare my clothes on.

Mary said:

Syphilis, gonorrhea, HIV, herpes; that ought to want to motivate anybody to practice safe sex.

Other women said that their desire not to become pregnant motivated them to practice safe sex. Sally was motivated by the need to care for her children.

She stated:

Because I have three children and I don't plan on dying until the Lord takes me.

A smaller number of women declared that they were motivated to practice safe sex because they had seen, first hand, the effects of HIV.

Kim said:

Things that motivate me – you see somebody outside on the streets and you see them with it and you see the effect that it's had on them and you look at them and you say that's something that you don't want to do so it motivates you to practice safe sex.

### Intervention components

When asked to describe what should be included in a videotape aimed at prevention of HIV within the African-American community, the most prevalent response among participants was to include personal experiences of people affected by HIV and AIDS. They believed that testimonials from those infected with HIV and sensational footage of the ravaging effects HIV and AIDS have on the human body would be most effective. To create a video that addresses HIV prevention, over half of the participants recommended sensationalism to garner the audience's attention. The purpose of the video would be to show the illness, pain, rejection, medication regimen, and years of life lost among those infected.

Fonda, a 24 year old GED preparer with 2 years of college said:

To make them watch it, show the blood, the guts, the pus, the sores, the relationships...

Sally said:

Show them how the body deteriorates from having AIDS. Show them everything else in the world that they are going to miss out on if they don't take care of themselves.

Another participant said:

You need to let them see how these people are just in so much pain and rejection, and not having the finances and things. In order to live like the guy that's an athlete [Magic Johnson], you know to live by the medication, how people are going to other places to get it.

A variety of other suggestions were given including the use of popular culture in the forms of rap and gospel music videos, productions and concerts and creating a Surgeon General's warning against unsafe sex [much like what has been done with tobacco],. Ceah, a 25 year old health care worker said:

Drum them in any way you can. If they like rap, rap it to them. If they like gospel, you sing it to them. If prayer is what it takes, you pray it to them. By any means necessary, you get your message across. Try all ways.

Other recommendations included developing a video in the form of a comedy-drama or a cartoon or using a campaign similar to the one utilized by Mothers Against Drunk Driving in which a person pre-HIV is shown followed by the person who has become ill post-HIV infection.

The participants also offered suggestions for recruiting African Americans into prevention programs where the videotape could be shown. The majority of women suggested the use of financial incentives including air conditioning units, fans, food, and amusement park tickets. The women recommended recruiting participants within the communities in which they live, going door-to-door if needed, and having such programs take place in community settings, such as schools and neighborhood centers. Other suggestions included having family friendly events that men would want to attend, thus ensuring women and children will follow.

Fonda believed that you have to give something to get people to participate. She said:

You know, people want something for something; nobody wants nothing for nothing. Nothing is free in this world.

Shirley, a GED student suggested community involvement:

Fundraisers, cooking things, get the community involved with you as the person that wants to get these things started. Once you get these things started, once you get the community, you got it.

Nilene suggested:

Just get it [word] out, it has to be let out in some kind of way, as far as radio stations, TVs, billboards. People in the communities, the main office, the campus, I mean everywhere, it has to be everywhere. It can't be in one spot, it has to come out, go out.

Veronica, a 25 year old youth coordinator with one year of college says:

If you get the men there, the women will come. A basketball tournament, yes, they like basketball and once you get the men there, the women are gonna come. Seriously, it's bad to say it like that but you're giving something. It's a sex symbol, yes, you understand what I'm saying, but it's a way to get them out there.

## Conclusion

Prior research has indicated the need to develop HIV intervention programs that target the socioeconomic contexts of women at risk for infection including cognitive or social cognitive, educational and/or skills building content-specific components. Women in the present study recommended that HIV/AIDS videotaped messages should be developed that highlight the sensational effects of the disease. Contrary to research indicating this method does not work [30-32] and based on the results of the research presented here, it may be worthwhile to field test a videotape that features HIV-positive people with groups of African Americans to evaluate the utility of such a teaching tool as they may have an essential role to play in AIDS prevention. Similar to our findings with African American men [33], the participants also recommended free, confidential testing in a community-based setting with the provision of incentives for testing and participation, findings that offer a first glimpse at what researchers and practitioners can do to create culturally relevant HIV prevention programs for African-American women. Although the findings are limited due to the small sample size, the use of convenience sampling and the location in the southern part of the United States, this research may provide a base for conducting larger studies among low-income African-American women. Before programs are developed, the barriers poor African-American women face on a daily basis should be addressed. Programs are not only needed to help negotiate these barriers, the barriers should also be included in program development. This may necessitate the involvement of various social service agencies as well as health educators and nursing and medical professionals. The women also need skills training to enhance their abilities to negotiate safer sex practices with their partners. If the tide of HIV and AIDS infection among African Americans is to be reduced, programs must incorporate culturally relevant contextual information presented to the target audience in a setting and in a manner that addresses their norms and beliefs and provides them the knowledge and skills needed to make correct decisions^2^.

Health professionals may wish to learn more about the barriers these women face and work with social service providers to address the issues most salient to the women before developing patient education materials for HIV/AIDS prevention. Methods that appeal to the target audience should be devised but nursing professionals should remember that low-income African-American women are a heterogeneous group. Interventions such as videotapes should be developed to have a wide appeal yet have contextual, cultural, and gender specificity and it is important to remember that it is best to educate women within a community-based setting. Resources are needed to identify, recruit and retain African American women into HIV intervention programs.

## Competing interests

The author(s) declare that they have no competing interests.

## Contributors

EJE, AFM and GOO conceived and designed the study. EJE, AFM, RJP jointly planned and executed the data analyses. EJE wrote the paper with assistance from AFM, RJP, GOO and NIO.
